# Phylogeographic and Ecological Insights Into the Evolutionary History of the Grass‐ and Sedge‐Specializing Deltocephalinae Leafhoppers

**DOI:** 10.1002/ece3.72857

**Published:** 2026-01-05

**Authors:** Lili Tian, Liangchenyu Shan, Hui Zhang, Shuyu Wei, Zhengnan Li, Xinghu Qin, Bin Zhang

**Affiliations:** ^1^ College of Life Sciences & Technology Inner Mongolia Normal University Hohhot China; ^2^ The School Affiliated of Inner Mongolia Normal University Hohhot China; ^3^ School of Ecology and Nature Conservation Beijing Forestry University Beijing China; ^4^ International Machine Learning Laboratory for Biodiversity Research Beijing Forestry University Beijing China; ^5^ Capital Biodiversity Conservation Institute Beijing China; ^6^ College of Horticulture and Plant Protection Inner Mongolia Agricultural University Hohhot China; ^7^ Key Laboratory of Biodiversity Conservation and Sustainable Utilization for College and University of Inner Mongolia Autonomous Region Hohhot China; ^8^ Key Laboratory of Infinite‐Dimensional Hamiltonian System and Its Algorithm Application Ministry of Education Hohhot China

**Keywords:** Bayesian phylogeographic reconstructions, climate change, Deltocephalinae, ecological niche modeling, grass/sedge‐specialization

## Abstract

Grass‐ and sedge‐specializing species comprise approximately one‐third of the tribes in the Deltocephalinae subfamily, making them an integral part of grassland ecosystems in Mongolia, where grasslands and arid rangelands dominate. However, their evolutionary and ecological dynamics remain poorly understood. We sampled 22 sites across Mongolia and identified 37 species from 16 genera of Deltocephalinae, including 10 new records for Mongolia. These sites were specifically selected to focus on regions with high diversity and abundance of grass‐ and sedge‐specialized Deltocephalinae, based on previous surveys and accessibility for fieldwork. Seventeen of the 22 sites are concentrated in central and eastern Mongolia, representing key habitats rather than a fully random or uniform distribution across the country. Phylogenetic analyses supported the monophyly of grass‐ and sedge‐specializing tribes, and divergence dating placed their split from other Deltocephalinae at ~95.9 Ma, predating the origin of grasses and sedges (76–88 Ma). This suggests early host shifts or ecological flexibility, with subsequent diversification and dispersal coinciding with the expansion of grasslands. Ecological niche modeling revealed that the distribution of grass‐ and sedge‐specializing species contracted during the Last Glacial Maximum, likely due to colder, drier climates and predicts further contraction under future anthropogenic pressures such as overgrazing. This study provides the first comprehensive phylogeographic and ecological analysis of grass‐ and sedge‐specializing leafhoppers in Mongolia, offering new insights into their diversification and interactions with grassland ecosystems.

## Graphical abstract

Phylogenetic reconstructions and morphological analysis robustly support the monophyletic status of tribes specializing in grass and sedge habitats. Grass‐ and sedge‐specializing groups diverged from nonspecializing groups around 95.85 million years ago (Ma) during the Cretaceous period, preceding the origin of grasses and sedges (76–88 Ma), indicating early host shifts or ecological flexibility. Distribution patterns show a contraction of grass‐ and sedge‐specializing species during the Last Glacial Maximum (LGM), potentially due to colder and drier climates. Anticipated habitat contraction is attributed to anthropogenic factors such as overgrazing, suggesting ongoing environmental pressures on these species.

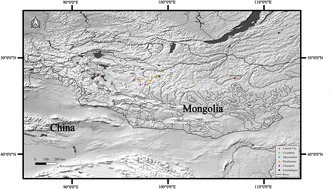

## 1 Introduction

Deltocephalinae, the largest subfamily of Cicadellidae (leafhoppers), includes 6683 valid species and 923 genera distributed across diverse ecosystems, from arid grasslands and shrublands to tropical rainforests (Zahniser and Dietrich 2010, 2013). Many species are economically important as vectors of plant diseases that cause major agricultural losses. While most feed on dicotyledonous plants, about one‐third of tribes specialize in grasses and sedges, particularly in grassland ecosystems (Zahniser and Dietrich 2010, 2013). This specialization is considered a phylogenetically conserved trait within Deltocephalinae (Zahniser and Dietrich 2010). However, few studies have focused on this group, particularly in Mongolia, which contains part of the largest natural grassland system in the world (Zahniser and Dietrich 2010, 2013).

Previous studies have mostly focused on the morphology and phylogenetic relationships within Deltocephalinae (Hamilton 1975; Emeljanov 1999; Dmitriev 2002; Dietrich 2002; Zahniser and Dietrich 2010, 2013), leaving the evolutionary and ecological dynamics relative to the origin of grasses (74–82 Ma; Christin et al. 2014), sedges (77–89 Ma; Spalink et al. 2016), and the subsequent spread of grasslands (15–5 Ma; Strömberg 2011) could provide critical insights into their evolutionary association with grass‐dominated habitats. These events likely influenced the diversification of these species. However, due to limited fossil evidence and methodological constraints in earlier studies, molecular dating of Deltocephalinae lineages has remained challenging. Recent advances in secondary calibration methods (Liu et al. 2019) now provide improved estimates of divergence times.

Mongolia hosts the largest continuous natural grassland ecosystem shared with China (Pfeiffer et al. 2018), supporting 3127 vascular plant species (Urgamal et al. 2014) and a rich insect fauna (Bayartogtokh et al. 2016). However, research on Deltocephalinae in Mongolia remains limited. Understanding the evolutionary history and distribution in the context of Mongolia's unique environment can reveal how geography, climate, and host specialization interact.

This study integrates phylogenetic analyses, molecular dating, Bayesian phylogeographic diffusion, and ecological niche modeling (ENM) to address these gaps. Specifically, we focus on grass‐ and sedge‐specializing Deltocephalinae in Mongolia to: (i) reconstruct phylogeographic structure, (ii) estimate the timing of their divergence relative to grasses and sedges, (iii) infer dispersal patterns and range, (iv) predict future habitat suitability. These approaches contribute to a deeper understanding of the evolutionary and ecological dynamics of this specialized lineage.

## 2 Materials and Methods

### 2.1 Sample Collection and Identification

**FIGURE 1:**
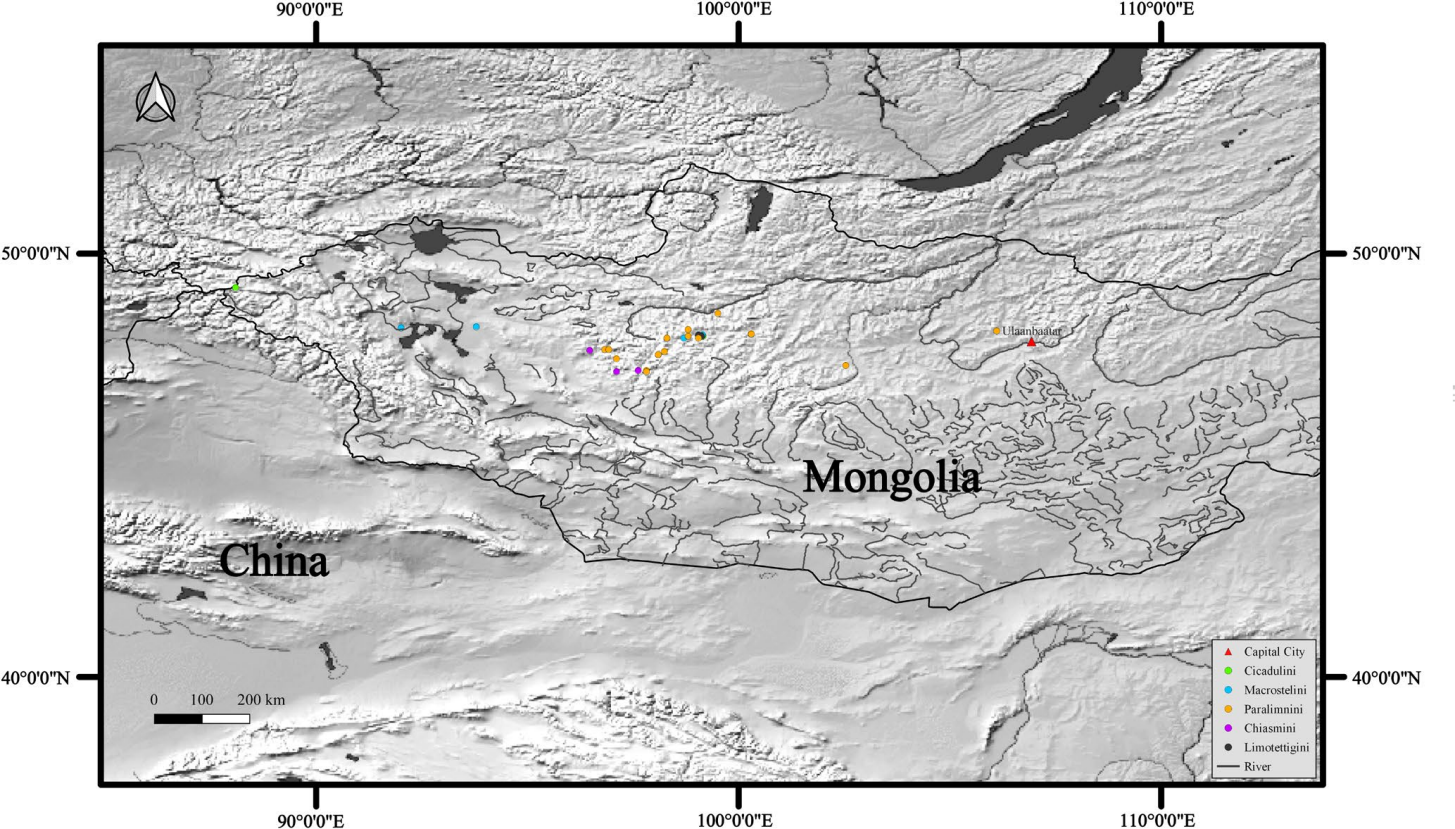
Geographic sampling of grass‐ and sedge‐specializing species of Deltocephalinae in Mongolia. The sampling sites are plotted on a digital elevation map of the studied areas, and colored by seven regions: green indicating Cicadulini; blue indicating Macrostelini; orange indicating Paralimnini; purple indicating Chiasmini; black indicating Limotettigini.

A total of 80 individuals representing the subfamily Deltocephalinae were collected from 22 sites across Mongolia (Table S1; Figure 1). Specimens were preserved in 95% ethanol and stored at −20°C before being deposited at Inner Mongolia Normal University. All collection and handling procedures were approved by the Animal Care and Use Committee of CIB 144 (Permit Number: CIB‐20121220A). Detailed information on specimen collection sites and corresponding sequences is provided in Table S1.

**FIGURE 2:**
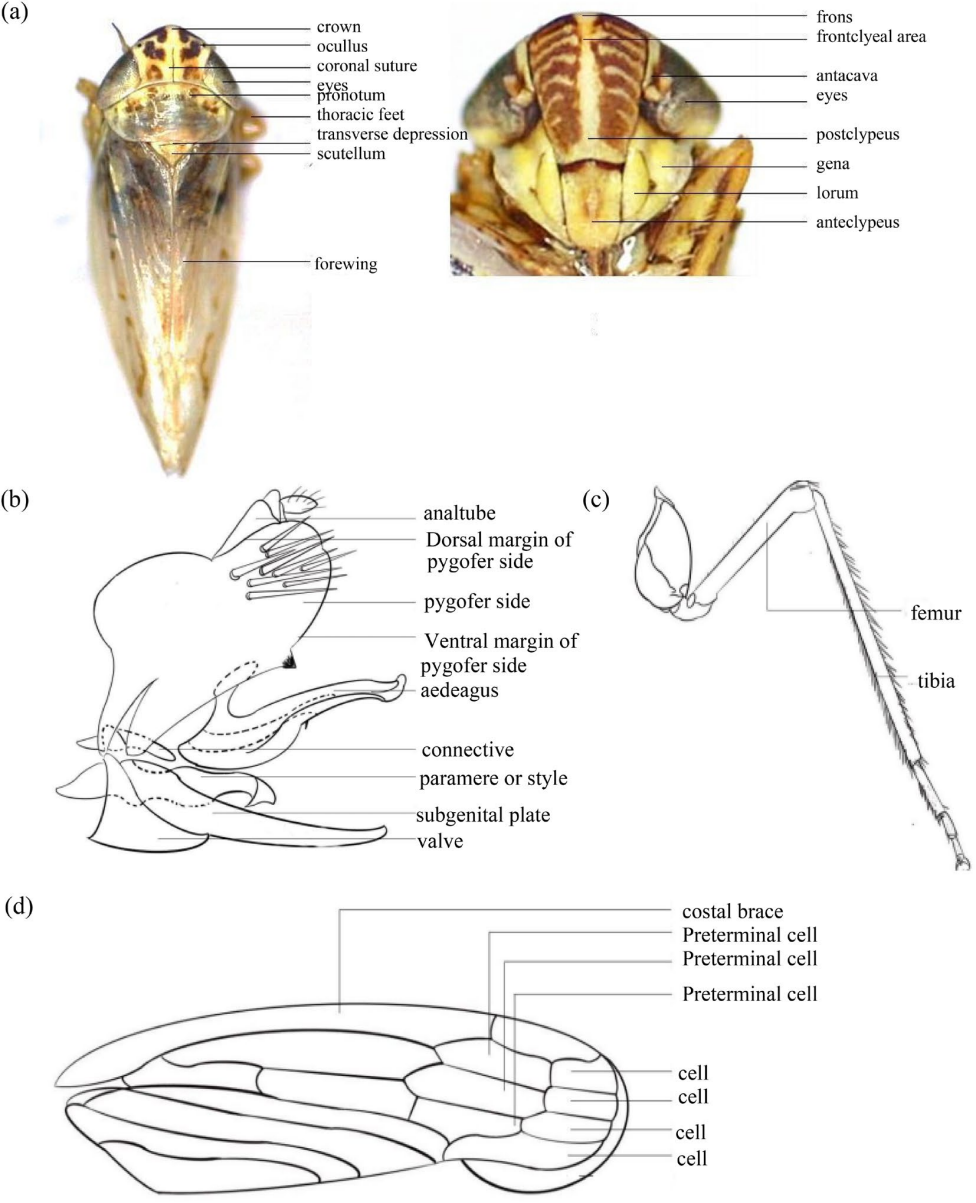
Morphological characters used for Deltocephalinae species identification. (a) Body structure and head of Deltocephalinae; (b) Adult male genitalia of Deltocephalinae; (c) Hind femur macrosetae structure of Deltocephalinae; (d) Forewing of Deltocephalinae.

Species identification was performed using a combination of morphological characteristics and molecular barcoding. Morphological identification followed standard taxonomic keys and methods described in Dietrich (1999), Dietrich et al. (2001) and Zahniser and Dietrich (2008). For molecular identification, we conducted BLAST searches to match the obtained sequences with previously published sequences in GenBank, enabling taxonomic classification. The morphological characters utilized in this analysis are illustrated in Figure 2.

### 2.2 DNA Extraction and Sequence Processing

The entire bodies of the 80 collected specimens (excluding abdomens) were shipped to Tsingke Biotechnology (Beijing, China) for DNA extraction. Total genomic DNA was extracted following the cetyltrimethylammonium bromide (CTAB) method (Reineke et al. 1998). The mitochondrial cytochrome c oxidase subunit I (COI) gene was amplified for all specimens. The resulting sequences were deposited in Dryad (http://datadryad.org/stash/share/JqzL5GsIRQ17wgBVGjLsDfOVjYutnbzHXiJFBNEKAIg, for reviewer).

All novel COI sequences were assembled and edited using SeqManII (DNASTAR, Madison, WI, USA) and translated into amino acid sequences using MEGA v7.0 (Kumar et al. 2016) to verify sequence accuracy and check for stop codons. Two outgroup sequences from other Deltocephalinae tribes (*Drabescus ineffectus* Walker, 1858 and *Fieberiella septentrionalis* Wagner, 1963) were retrieved from GenBank (accession numbers MT527188 and MW078430, respectively). Sequence alignment was performed using Clustal X v2.0 (Larkin et al. 2007), and no insertions or deletions (indels) were observed in the COI matrices.

### 2.3 Phylogenetic Analysis

Phylogenetic relationships were reconstructed using Bayesian inference (BI) and maximum likelihood (ML) methods based on the COI sequences. The dataset was partitioned into three subsets corresponding to the first, second, and third codon positions of the COI gene. PartitionFinder v2.1.1 (Lanfear et al. 2012) was used to determine the best‐fit partitioning scheme and nucleotide substitution models under the Bayesian information criterion (BIC).

For BI, analyses were conducted using MrBayes v3.2.6 (Ronquist et al. 2012), with four independent runs of 20 million generations, sampling every 5000 generations. A 25% burn‐in phase was applied, and a 50% majority‐rule consensus tree was constructed from the combined runs. Posterior probabilities (PP) ≥ 0.95 were considered strong support (Erixon et al. 2003; Huelsenbeck and Rannala 2004). ML analyses were performed using IQ‐TREE v1.6.7 (Nguyen et al. 2015) with 5000 ultrafast bootstrap (UFBoot) replicates. Nodes with UFBoot values ≥ 95 were considered robust (Minh et al. 2013). Final phylogenetic trees were visualized using FigTree v1.4.4 (Rambaut 2016), and additional edits were made in Microsoft PowerPoint (2010).

### 2.4 Divergence Times Estimation

Divergence times were estimated using BEAST v1.8.4 (Drummond and Rambaut 2007) under two models: a relaxed log‐normal clock and a strict clock. Results from both approaches were compared. Given the scarcity of reliable fossil evidence for Deltocephalinae, secondary calibration points were used for robust divergence time estimation.

We incorporated six calibration points, each with a normal distribution of probability densities, based on previous studies (Cao et al. 2022):

C1: 52.12 ± 2.3 Ma, representing the most recent common ancestor (MRCA) of the tribe Paralimnini.

C2: 55.14 ± 3 Ma, representing the MRCA of the tribe Chiasmini.

C3: 33.41 ± 2.19 Ma, representing the MRCA of the tribe Limotettigini.

C4: 47.16 ± 2.7 Ma, representing the MRCA of the tribe Cicadulini.

C5: 48.03 ± 2.05 Ma, representing the MRCA of the tribe Fieberiellini.

C6: 66.84 ± 2.3 Ma, representing the MRCA of the tribe Drabescini.

These calibrations were selected based on consistent use in previous large‐scale phylogenetic analyses of Deltocephalinae, where dense sampling of taxa and morphological characters has demonstrated improved resolution and reliable divergence estimates (Cao et al. 2022). While direct fossil evidence is scarce, these secondary calibrations provide a pragmatic framework for time calibration.

The Markov Chain Monte Carlo (MCMC) analysis was run for 50 million generations, with sampling every 3000 generations. Convergence and effective sampling size (ESS > 200) were assessed using Tracer v1.7.1. Divergence time estimates were summarized using TreeAnnotator v2.6.2 and visualized in FigTree v1.4.4.

### 2.5 Phylogeographic Diffusion in Continuous Space

Spatial diffusion across Mongolia was modeled using a Bayesian phylogeographic approach implemented in BEAST v1.10.4 (Suchard et al. 2018). This method allows continuous diffusion of genealogies without arbitrarily dividing species ranges into discrete operational areas. Sampling locations were treated as bivariate traits (latitude and longitude).

To determine the best‐fit diffusion model, marginal‐likelihood estimation via generalized stepping‐stone sampling was performed, comparing three relaxed random walk models (RRW) and a time‐homogeneous Brownian motion (BM) process. A constant‐size coalescent tree prior and a strict molecular clock (with the divergence time of grass‐ and sedge‐specializing groups set to 95.5 Ma) were applied.

The MCMC analysis was run for 50 million generations, sampling every 3000 generations. Convergence was assessed in Tracer v1.7.1. Phylogeographic reconstructions were visualized using SpreaD3 (Bielejec et al. 2016).

### 2.6 Ecological Niche Modeling

ENM was performed to predict the suitable habitat range of grass‐ and sedge‐specializing Deltocephalinae during six climatic epochs: the mid‐Pliocene warm period (MPWP, 3.205 Ma), Last Interglacial (LIG, 0.14–0.12 Ma), Last Glacial Maximum (LGM, 0.026–0.019 Ma), present‐day, and two future scenarios (2050s and 2070s). For future predictions, we used two contrasting climate scenarios from the CMIP6 framework: SSP1‐2.6 (low‐emission pathway) and SSP5‐8.5 (high‐emission pathway), which represent alternative trajectories of anthropogenic climate change.

Climatic data for each epoch were sourced from WorldClim (Hijmans et al. 2005) and PaleoClim (Brown et al. 2017). A total of 19 bioclimatic variables were initially compiled, but variables with Pearson correlation coefficients (|*r*| > 0.8) were excluded to reduce multicollinearity, leaving eight variables for analysis (Table S2). Occurrence data were filtered to ensure spatial independence by rarefying sampling localities at a 30‐km distance using SDMtoolbox v2.0 in ArcGIS v10.2 (Brown et al. 2017). Twenty‐two spatially independent occurrence points were retained.

ENMs were constructed using MAXENT v3.4.4 (Phillips et al. 2006) with 70% of the data used for training and 30% for testing. Models were run with 100 subsample replicates and 1000 iterations. The performance of each model was evaluated using the area under the receiver operating characteristic curve (AUC). The AUC value ranges from 0 to 1, with values closer to 1 indicating higher model credibility (Xu et al. 2015). The following classification is generally accepted: 0–0.5 (fail), 0.5–0.7 (poor), 0.7–0.8 (fair), 0.8–0.9 (good), and 0.9–1.0 (excellent) (Hanley and McNeil 1982). A logistic threshold of 10% training presence was applied to define the minimum probability of suitable habitats. In addition, two potential habitat types are classified as follows: high habitat suitability (0.8 < *p* ≤ 1), medium habitat suitability (0.6 ≤ *p* < 0.8). ENM outputs were visualized and edited using ArcGIS v10.2.

## 3 Results

### 3.1 Sequence Characteristics and Species Identification of Deltocephalinae

A total of 80 mitochondrial COI gene sequences were generated, representing 37 species of Deltocephalinae, including 10 species recorded for the first time in Mongolia. The dataset consisted of 327 conserved sites, 358 variable sites, 66 singleton sites, and 291 parsimony‐informative sites.

**FIGURE 3:**
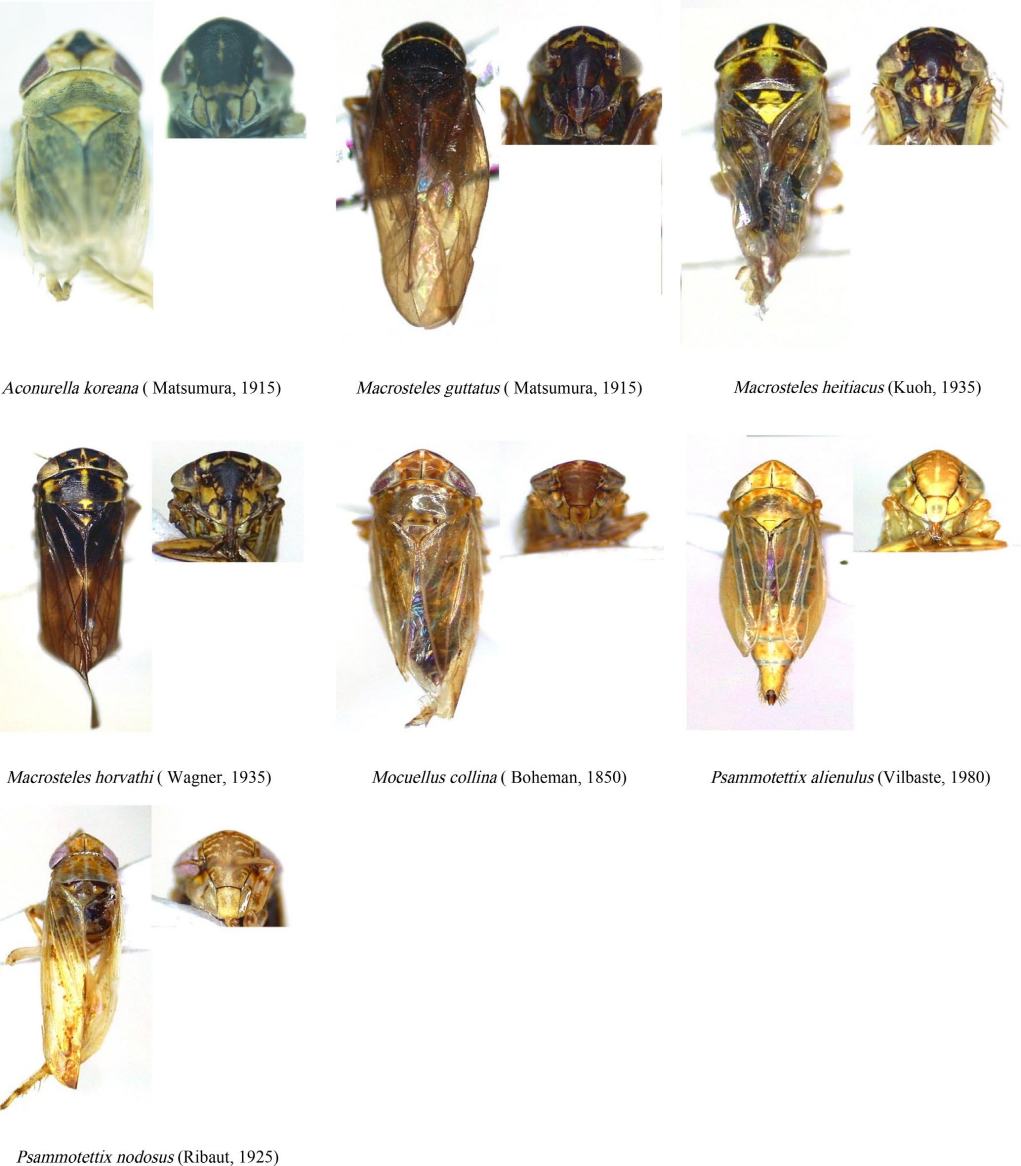
Seven new species records of Deltocephalinae in Mongolia. Photoed by Guihua Liu.

Sequences aligned using BLAST on NCBI showed high identity (> 90%) with species within the subfamily Deltocephalinae. Morphological identification, based on key characters from the head, thorax, abdomen, male genitalia, and wing structures (Figure 2), corroborated the molecular results. The combined molecular and morphological analyses identified 37 species from 16 genera. Among the sequences, 79 unique haplotypes were detected, with only one haplotype being shared between individuals of the same species. Based on both molecular and morphological data, 10 new records of Deltocephalinae species in Mongolia were identified: *Mocuellus inepta* Emeljanov, 1964, *Ophiola corniculus* Marshall, 1866, *Macrosteles horvathi* Wagner, 1935, *Destitutus kungurtuki* Vilbaste, 1980, *Psammotettix alienulus* Vilbaste, 1980, *Mocuellus collina* Boheman, 1850, *Macrosteles guttatus* Matsumura, 1915, *Macrosteles heitiacus* Kuoh, 1981, *Aconurella koreana* Matsumura, 1915, and *Psammotettix nodosus* Ribaut, 1925 (Figure 3).

### 3.2 Phylogenetic Analyses of Grass‐ and Sedge‐Specializing Species of Deltocephalinae in Mongolia

**FIGURE 4:**
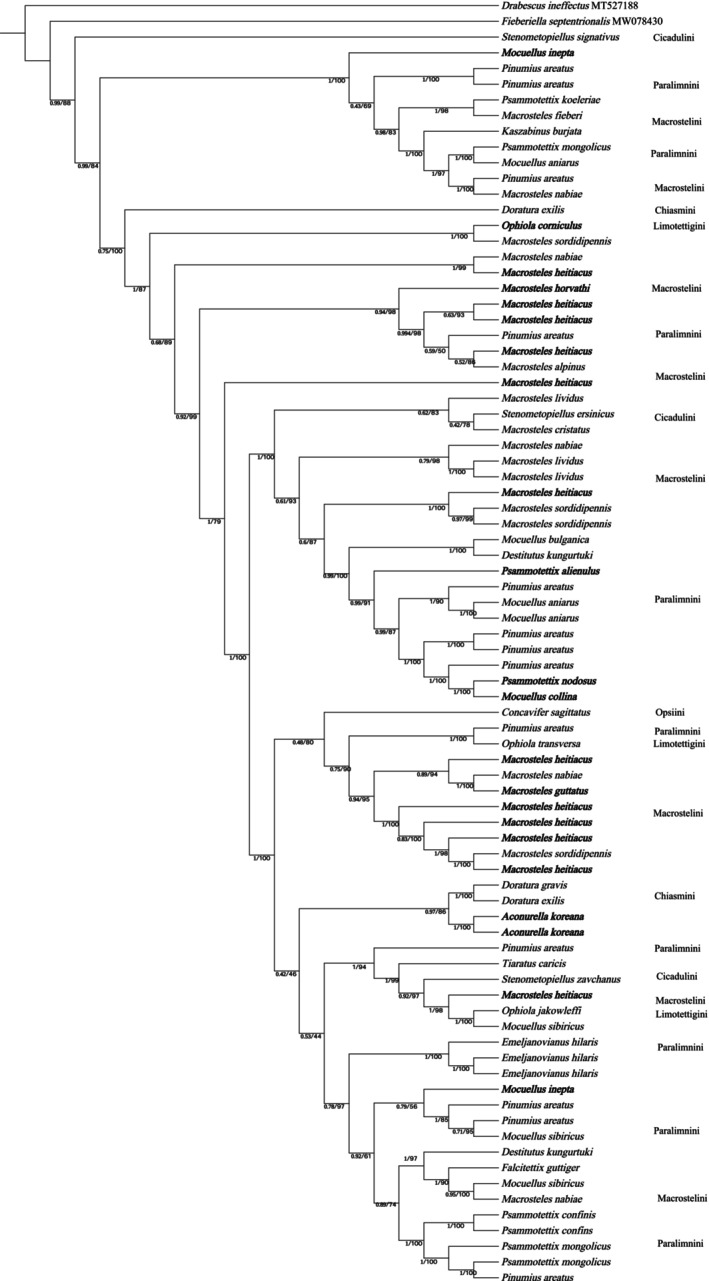
Hypothesized phylogenetic relationships of grass‐ and sedge‐specializing species of Deltocephalinae in Mongolia, illustrated by the 50% majority‐rule consensus tree resulting from partitioned Bayesian analyses. Bayesian posterior probabilities and maximum likelihood bootstrap values are shown.

BI and ML analyses produced congruent topologies, differing only in a few minor nodes. The BI tree, annotated with PP and UFBoot values from the ML analysis, is shown in Figure 4. Two outgroup species (*D. ineffectus* and *F. septentrionalis*), representing nongrass‐ and nonsedge‐specializing tribes (*Drabescini* and *Fieberiellini*), were used to root the tree.

The monophyly of the grass‐ and sedge‐specializing group was strongly supported (PP = 0.99). However, the monophyly of the six tribes (*Chiasmini*, *Opsiini*, *Paralimnini*, *Macrostelini*, *Limotettigini*, and *Cicadulini*) could not be resolved, likely due to the limited sequence length analyzed. This study presents, for the first time, a detailed phylogenetic reconstruction of grass‐ and sedge‐specializing Deltocephalinae species in Mongolia.

### 3.3 Diversification Timeframe of Grass‐ and Sedge‐Specializing Lineages

**FIGURE 5:**
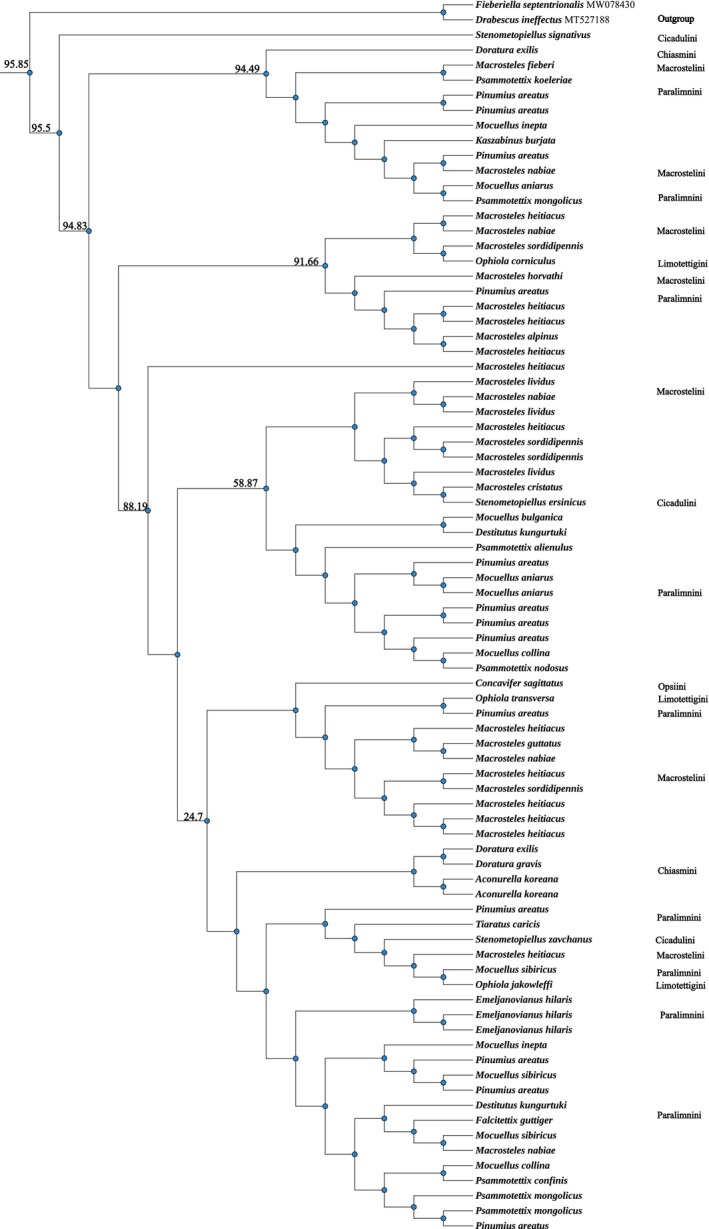
Divergence times estimate of the major lineages within grass‐ and sedge‐specializing group inferred from mtDNA COI data.

Divergence time estimates for the grass‐ and sedge‐specializing lineages are shown in Figure 5. The split between grass‐/sedge‐specializing species and their nonspecialist outgroups occurred approximately 95.85 Ma during the Cretaceous. Within the grass‐ and sedge‐specializing clade, divergence among the six tribes occurred around 95.5 Ma. Subsequent divergence events included: (1) Chiasmini and Macrostelini: ~93.82 Ma; (2) Paralimnini and Macrostelini: ~92.27 Ma; (3) Limotettigini and Macrostelini: ~88.77 Ma. The estimated mean substitution rate for grass‐ and sedge‐specializing Deltocephalinae was 3.51% per site per million years (95% HPD: 2.95%–4.18%).

### 3.4 Spatiotemporal Diffusion of Grass‐ and Sedge‐Specializing Species in Mongolia

**FIGURE 6:**
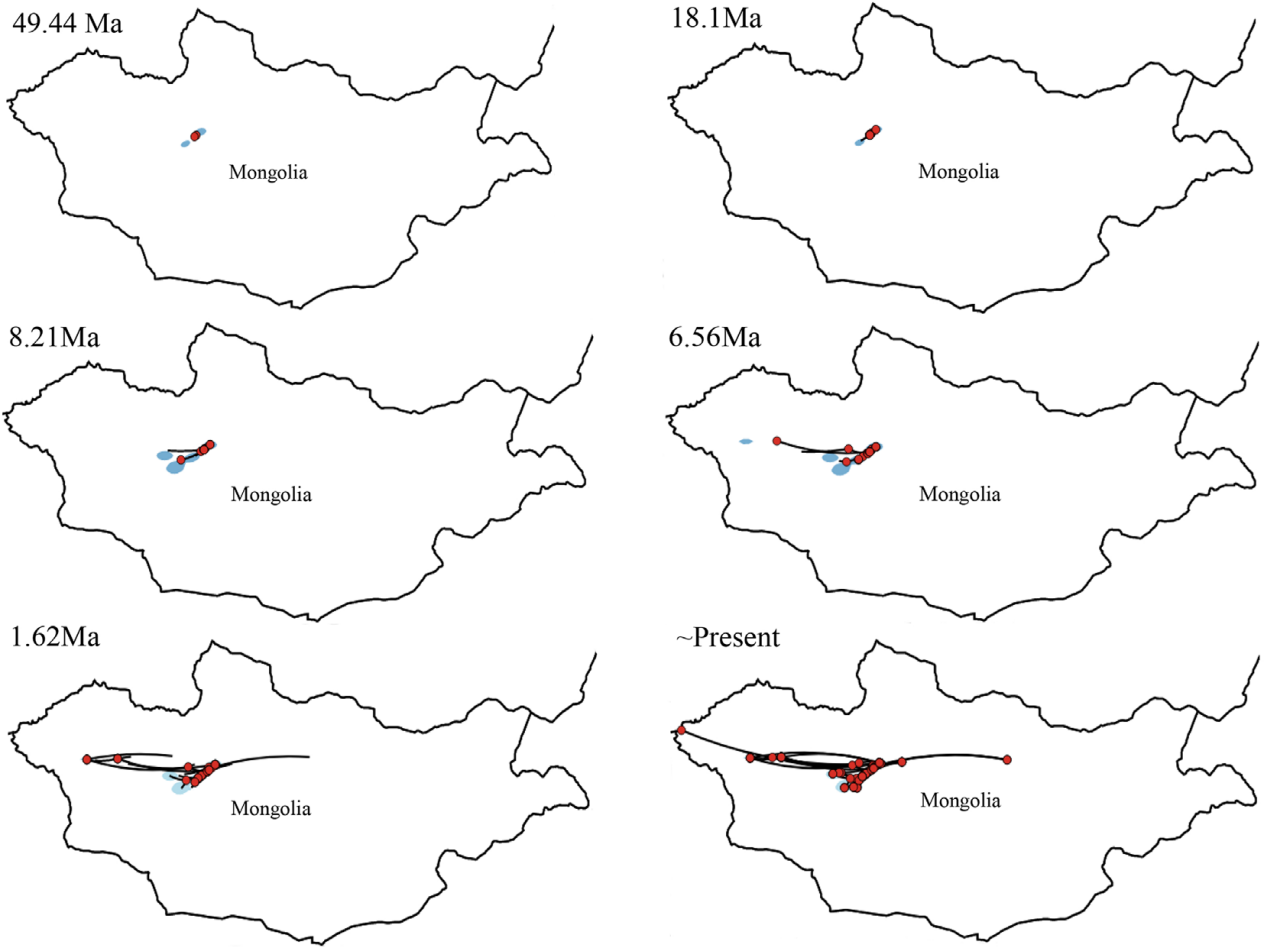
Spatiotemporal reconstruction of the geographic dispersal of grass‐ and sedge‐specializing group using the Bayesian phylogeographic method. Six snapshots indicate the distributed directions.

We evaluated the fit of three RRW models and a time‐homogeneous BM model to the spatiotemporal diffusion of six grass‐ and sedge‐specializing tribes within the Deltocephalinae subfamily. Model selection based on Bayesian factors (BF) and effective sample sizes (ESS) favored the BM model for all six tribes (all ESS > 200; report specific BF values for transparency). This suggests that the dispersal of these tribes across Mongolia can be approximated by a random walk process. Figure 6 illustrates the inferred dispersal history based on the BM model, highlighting key phylogeographic events. At approximately 40.74 million years ago (Ma), the species *Stenometopiellus signativus*, belonging to the tribe Cicadulini, initiated dispersal from Chair County in Arkhangai Province, Mongolia. Subsequent dispersal events show a westward trend, reaching areas occupied by Limotettigini (approx. 18.1 Ma, exemplified by *O. corniculus*), Chiasmini (approx. 8.21 Ma), and Macrostelini (approx. 6.56 Ma, exemplified by *M. heitiacus*). An eastward and southward shift is observed around 1.62 Ma, reaching areas occupied by some Paralimnini species. The contemporary distribution of *Stenometopiellus zavchanus* and *Pinumius areatus*, representing tribes Cicadulini and Paralimnini, respectively, completes the comprehensive phylogeographic profile of this unique Deltocephalinae assemblage in Mongolia. These dispersal events suggest a complex pattern of range expansion and contraction over time. Further research is needed to understand the drivers of these westward and subsequent eastward dispersal patterns.

### 3.5 Temporal Changes in Suitable Habitat Distribution

Maximum entropy (MaxEnt) species distribution models (SDMs) were constructed using eight noncollinear (*r* < 0.8) climate variables (Table S2; eight variables for the Middle Paleolithic‐Warm Period [MPWP]) to predict the suitable distributional areas of grass/sedge‐specializing Deltocephalinae in Mongolia during five epochs: LIG, LGM, present, and future projections (2050s, 2070s). All models exhibited high predictive performance (AUC > 0.90). Variable importance varied across epochs. For epochs excluding the LIG, annual mean temperature (BIO1) consistently contributed most (mean relative contribution: 30.38%), followed by mean diurnal range (BIO2) in future projections and precipitation of the wettest quarter (BIO16) in the MPWP, LGM, and present. In contrast, the LIG model was primarily driven by precipitation of the driest quarter (BIO17, 55.48%) and mean temperature of the wettest quarter (BIO8, 16.53%). This highlights the significant role of both temperature and precipitation in shaping the species' distribution across different climatic periods.

**FIGURE 7:**
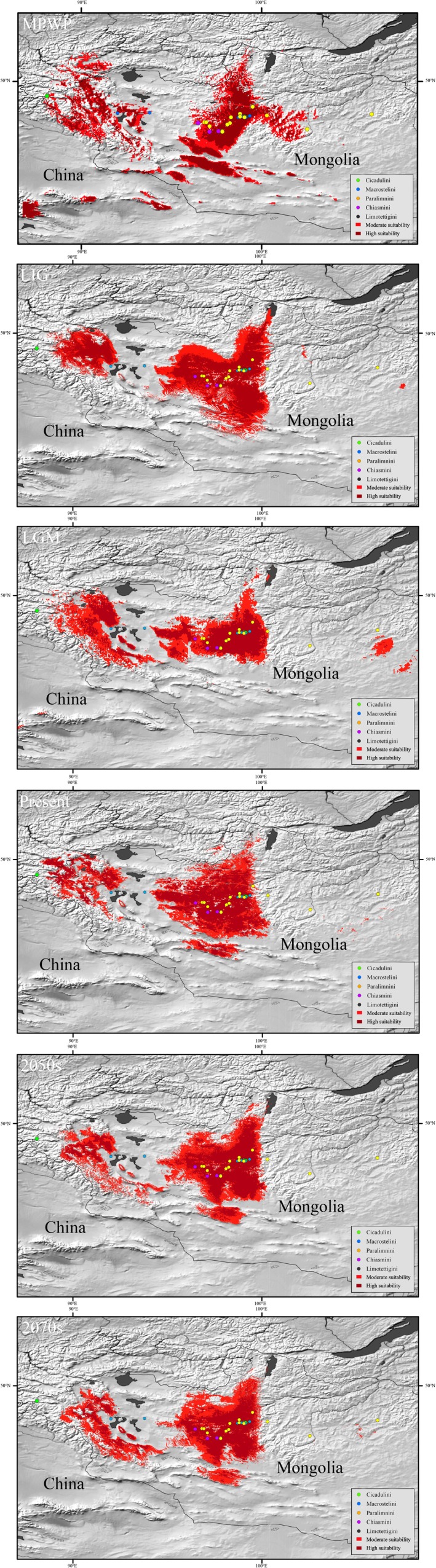
Projected distributions of grass‐ and sedge‐specializing group during LIG, LGM, MPWP, 2050s, 2070s, and Present‐day. Dark red indicates high suitability (0.8 < suitable index ≤ 1) and light red indicates moderate suitability (0.6 ≤ suitable index < 0.8). The sampling sites are plotted on a digital elevation map of the studied areas, and colored by five regions: green indicating Cicadulini; blue indicating Macrostelini; orange indicating Paralimnini; purple indicating Chiasmini; black indicating Limotettigini.

Model predictions (Figure 7) largely align with current collection and literature records. Areas with high suitability (0.8 < suitable index ≤ 1) and moderate suitability (0.6 ≤ suitable index < 0.8) are distributed throughout the range, except for the easternmost locations of Paralimnini. The widest suitable range was predicted for the MPWP, while the largest area of suitable habitat was predicted for the LIG. During the LGM, the suitable range contracted but became more continuous compared to the LIG. The present‐day distribution more closely resembles the LIG than the LGM. Future projections suggest a slight contraction of suitable range compared to the present. Further investigation is needed to understand the specific climatic factors driving these changes and their potential impact on Deltocephalinae populations.

## 4 Discussion

Using phylogenetic reconstruction, molecular dating, Bayesian phylogeographic approaches and ENM, this study reports grass‐ and sedge‐specializing Deltocephalinae in Mongolia. We traced their divergence timeline relative to the origin and spread of grasses and sedges, reconstructed spatiotemporal diffusion patterns, and predicted their suitable habitats across six key climatic epochs. These findings shed new light on the evolutionary history of this diverse group and the combined influence of geography and past climate on their distribution. We also investigated the relationship between the flora and fauna in Mongolia. The results regarding the phylogeographic history should be interpreted with caution since mtDNA tells only one part of a potentially more complex story (Ballard and Whitlock 2004; Galtier et al. 2009). Nevertheless, finer scale sampling incorporating niche modeling methods and estimates of migration may be able to identify more precisely mechanisms of diversification. Future studies based on multiple nuclear loci are highly desirable, even though mtDNA will continue to be a mainstay of phylogeography (Zink and Barrowclough 2008; Barrowclough and Zink 2009).

### 4.1 Phylogeographic Structure of Grass/Sedge‐Specializing Deltocephalinae in Mongolia

This study builds upon the foundational work of Zahniser and Dietrich (2010, 2013), who reconstructed the phylogenetic relationships of the grass‐ and sedge‐specializing Deltocephalinae. Focusing on six tribes, we sequenced and analyzed 80 specimens and used two outgroup species. Consistent with previous studies, the monophyly of the grass‐ and sedge‐specializing group was strongly supported (PP = 0.99), confirming that grass‐ and sedge‐specialization is a phylogenetically conserved trait within Deltocephalinae (Dietrich 1999; Dietrich et al. 2001; Zahniser and Dietrich 2010).

While the monophyly of the six tribes was not fully resolved due to limited sequence length, the relationships among tribes were partially clarified. For instance, Macrostelini was found to be closely related to Chiasmini, with Limotettigini also showing a closer relationship to Chiasmini. These findings are consistent with previous phylogenetic analyses (Zahniser and Dietrich 2013) and highlight the need for finer‐scale sampling and additional molecular data to fully resolve the evolutionary relationships within this group.

### 4.2 The Timing of Divergence in Grass‐ and Sedge‐Specializing Deltocephalinae

The timing of divergence among grass‐ and sedge‐specializing tribes within Deltocephalinae has remained unexplored in previous studies due to the lack of reliable fossil calibrations. Using secondary calibration points, we estimated the divergence time between the grass‐ and sedge‐specializing group and their nonspecialist outgroups to be approximately 95.85 million years ago (Ma), during the Cretaceous. This predates the origin of grasses and sedges, which is estimated to have occurred between 74–82 Ma (Christin et al. 2014) and 77–89 Ma (Spalink et al. 2016). These findings suggest that the initial divergence of the grass‐ and sedge‐specializing group may have occurred before these host plants became widespread, potentially implying an early host switch or a period of ecological flexibility before specialization.

The divergence of grass‐ and sedge‐specializing tribes within the group followed a pattern consistent with the origin and spread of grasslands (15–5 Ma; Strömberg 2011). For example, the rapid diversification and dispersal around 8.21 Ma coincides with the expansion of grassland ecosystems. The extreme morphological adaptations observed in certain taxa, such as the elongated and flattened heads in Dorycephalini and Eupelicini, may reflect evolutionary responses to host specialization (Zahniser and Dietrich 2010). Together, these findings support the hypothesis that the spread of grasses and sedges exerted strong selective pressures, driving both host specialization and morphological diversification within Deltocephalinae.

### 4.3 Dispersal Dynamics of Host Plants and Grass‐/Sedge‐Specializing Species

Bayesian diffusion models indicate that dispersal of grass‐ and sedge‐specializing tribes began approximately 40.74 Ma, later than the MRCA (Cao et al. 2022). A rapid dispersal phase occurred around 8.21 Ma, coinciding with the global expansion of grasslands. This timing suggests the spread of grass‐dominated habitats may have facilitated the geographic expansion of this group, as they are known to feed primarily on grasses (Poaceae) and sedges (Cyperaceae).

During the Pleistocene, glacial–interglacial cycles, characterized by major climatic fluctuations, significantly influenced the distribution and genetic diversity of many species (Avise 2000; Hewitt 1996, 2000, 2004). Our ENM analyses revealed that the MPWP provided the broadest suitable range for grass‐ and sedge‐specializing species, followed by a stepwise westward dispersal from 6.56 to 1.62 Ma. By 1.62 Ma, the group had nearly reached its modern distribution, excluding the most extreme eastern and western locations.

It should be noted that our ENM incorporated only bioclimatic predictors and did not explicitly model host plant distributions or co‐occurrence. Thus, inferences about the role of host plants should be interpreted with caution. Future work that integrates host plant distribution data with leafhopper occurrences will be essential to test potential coevolutionary patterns and to refine ecological niche predictions.

### 4.4 Impact of Climate Change on Distribution

The LGM (0.026–0.019 Ma; Clark et al. 2009), characterized by low temperatures and aridity, caused significant contractions in the distribution of temperate‐adapted taxa (Hewitt 2004; Provan and Bennett 2008). Similarly, our results show that the suitable habitat for grass‐ and sedge‐specializing Deltocephalinae in Mongolia contracted during the LGM compared to the LIG. This contraction likely reflects the reduced availability of grasses and sedges under cold and dry conditions, which negatively impacted these species' feeding and oviposition hosts. Following the LGM, the suitable range expanded, consistent with warming climates and the recovery of grassland habitats.

Our future projections (2050s and 2070s) suggest a slight contraction in suitable habitat compared to the present day (1960–1990). This reduction may be attributed to ongoing environmental challenges, including climate change and anthropogenic pressures such as overgrazing, which has been identified as a significant factor affecting Mongolian grasslands (Pfeiffer et al. 2019). These findings underscore the vulnerability of grass‐ and sedge‐specializing species to both climatic and human‐induced changes. Meantime, our sampling was concentrated in western and central Mongolia, which reflects both habitat availability and fieldwork feasibility. This clustering may introduce some bias, and future work with broader geographic coverage (particularly in eastern and southern Mongolia) will help refine our phylogeographic inferences and model predictions.

## Author Contributions

**Lili Tian:** conceptualization (equal), data curation (equal), formal analysis (equal), investigation (equal), resources (equal), visualization (equal), writing – original draft (equal). **Liangchenyu Shan:** formal analysis (equal), investigation (equal), methodology (equal), resources (equal), writing – original draft (equal). **Hui Zhang:** data curation (equal), formal analysis (equal), investigation (equal), resources (equal). **Shuyu Wei:** data curation (equal), investigation (supporting), methodology (equal), resources (supporting), visualization (equal), writing – review and editing (equal). **Zhengnan Li:** investigation (equal), resources (equal), writing – original draft (equal). **Xinghu Qin:** methodology (equal), resources (equal), supervision (equal), validation (equal), writing – original draft (equal), writing – review and editing (equal). **Bin Zhang:** conceptualization (equal), data curation (equal), formal analysis (equal), funding acquisition (equal), investigation (equal), methodology (equal), project administration (equal), resources (equal), supervision (equal), writing – original draft (equal), writing – review and editing (equal).

## Conflicts of Interest

The authors declare no conflicts of interest.

## Supporting information


**Table S1:** Detailed information on 80 specimen collection sites and corresponding sequences.

**Table S2:** Eight variables for the Middle Paleolithic‐Warm Period.

## Data Availability

The resulting sequences were deposited in Dryad (Reviewer URL: https://datadryad.org/dataset/doi:10.5061/dryad.hhmgqnks6).
