# Supplementary material for: Phylogeographic and Ecological Insights Into the Evolutionary History of the Grass‐ and Sedge‐Specializing Deltocephalinae Leafhoppers
Source: Ecol Evol. 2026 Jan 5;16(1):e72857. doi: 10.1002/ece3.72857 (PMC12771680; doi:10.1002/ece3.72857)
Supplement: Supplementary file 1 — Table S1: Detailed information on 80 specimen collection sites and corresponding sequences. [file ECE3-16-e72857-s001.docx]

| Species | Geographical origin | Collection Date | Altitude | Latitude | Longitude | Genebank Accession Number |
| --- | --- | --- | --- | --- | --- | --- |
| *Aconurella koreana* | Bayanchandmani County,Tuv Province, Mongolia | 2019/8/9 | 1835 | 96.470 | 47.706 |  |
| *Aconurella koreana* | Bayanchandmani County,Tuv Province, Mongolia | 2019/8/9 | 1835 | 96.470 | 47.706 |  |
| *Destitutus kungurtuki* | Tsetserleg County, Arkhangai Province, Mongolia | 2019/8/2 | 1782 | 100.297 | 48.107 |  |
| *Destitutus kungurtuki* | Tsetserleg County, Arkhangai Province, Mongolia | 2019/8/2 | 1782 | 100.297 | 48.107 |  |
| *Doratura exilis* | Otgun County, Zavkhan Province, Mongolia | 2019/8/7 | 2149 | 97.620 | 47.240 |  |
| *Doratura exilis* | Otgun County, Zavkhan Province, Mongolia | 2019/8/7 | 2149 | 97.620 | 47.240 |  |
| *Doratura gravis* | Otgun County, Zavkhan Province, Mongolia | 2019/8/7 | 2380 | 97.112 | 47.215 |  |
| *Falcitettix guttiger* | Huta Gwendur County,Bulgan Province, Mongolia | 2018/7/28 | - | - | - |  |
| *Kaszabinus burjata* | Otgun County, Zavkhan Province, Mongolia | 2019/8/7 | 2144 | 97.820 | 47.210 |  |
| *Macrosteles alpinus* | Chair County, Arkhangai Province, Mongolia | 2019/8/3 | 2248 | 99.122 | 48.050 |  |
| *Macrosteles cristatus* | Chair County, Arkhangai Province, Mongolia | 2019/8/4 | 2136 | 99.049 | 48.077 |  |
| *Macrosteles fieberi* | Chair County, Arkhangai Province, Mongolia | 2019/8/5 | 2224 | 98.700 | 48.010 |  |
| *Macrosteles heitiacus* | Lberking County, Zavkhan Province, Mongolia | 2019/8/10 | 1122 | 93.618 | 48.273 |  |
| *Macrosteles heitiacus* | Lberking County, Zavkhan Province, Mongolia | 2019/8/10 | 1122 | 93.618 | 48.273 |  |
| *Macrosteles heitiacus* | Lberking County, Zavkhan Province, Mongolia | 2019/8/10 | 1122 | 93.618 | 48.273 |  |
| *Macrosteles heitiacus* | Lberking County, Zavkhan Province, Mongolia | 2019/8/10 | 1122 | 93.618 | 48.273 |  |
| *Macrosteles heitiacus* | Lberking County, Zavkhan Province, Mongolia | 2019/8/10 | 1122 | 93.618 | 48.273 |  |
| *Macrosteles heitiacus* | Lberking County, Zavkhan Province, Mongolia | 2019/8/10 | 1122 | 93.618 | 48.273 |  |
| *Macrosteles heitiacus* | Chair County, Arkhangai Province, Mongolia | 2019/8/4 | 2136 | 99.049 | 48.077 |  |
| *Macrosteles heitiacus* | Chair County, Arkhangai Province, Mongolia | 2019/8/4 | 2136 | 99.049 | 48.077 |  |
| *Macrosteles heitiacus* | Chair County, Arkhangai Province, Mongolia | 2019/8/4 | 2136 | 99.049 | 48.077 |  |
| *Macrosteles heitiacus* | Chair County, Arkhangai Province, Mongolia | 2019/8/4 | 2136 | 99.049 | 48.077 |  |
| *Macrosteles heitiacus* | Chair County, Arkhangai Province, Mongolia | 2019/8/4 | 2136 | 99.049 | 48.077 |  |
| *Macrosteles heitiacus* | Chair County, Arkhangai Province, Mongolia | 2019/8/4 | 2136 | 99.049 | 48.077 |  |
| *Macrosteles horvathi* | Chair County, Arkhangai Province, Mongolia | 2019/8/5 | 2129 | 99.050 | 48.078 |  |
| *Macrosteles lividus* | Ergan County, Khovd Province, Mongolia | 2019/8/11 | 1180 | 91.900 | 48.246 |  |
| *Macrosteles lividus* | Ergan County, Khovd Province, Mongolia | 2019/8/11 | 1180 | 91.900 | 48.246 |  |
| *Macrosteles lividus* | Ergan County, Khovd Province, Mongolia | 2019/8/11 | 1180 | 91.900 | 48.246 |  |
| *Macrosteles nabiae* | Chair County, Arkhangai Province, Mongolia | 2019/8/4 | 2136 | 99.049 | 48.077 |  |
| *Macrosteles nabiae* | Ergan County, Khovd Province, Mongolia | 2019/8/11 | 1180 | 91.900 | 48.246 |  |
| *Macrosteles nabiae* | Ergan County, Khovd Province, Mongolia | 2019/8/11 | 1180 | 91.900 | 48.246 |  |
| *Macrosteles nabiae* | Ergan County, Khovd Province, Mongolia | 2019/8/11 | 1180 | 91.900 | 48.246 |  |
| *Macrosteles nabiae* | Ergan County, Khovd Province, Mongolia | 2019/8/11 | 1180 | 91.900 | 48.246 |  |
| *Macrosteles sordidipennis* | Chair County, Arkhangai Province, Mongolia | 2019/8/5 | 2129 | 99.050 | 48.078 |  |
| *Macrosteles sordidipennis* | Chair County, Arkhangai Province, Mongolia | 2019/8/4 | 2136 | 99.049 | 48.077 |  |
| *Macrosteles sordidipennis* | Chair County, Arkhangai Province, Mongolia | 2019/8/5 | 2129 | 99.050 | 48.078 |  |
| *Macrosteles sordidipennis* | Chair County, Arkhangai Province, Mongolia | 2019/8/4 | 2136 | 99.049 | 48.077 |  |
| *Mocuellus aniarus* | Otgun County, Zavkhan Province, Mongolia | 2019/8/6 | 2668 | 98.101 | 47.610 |  |
| *Mocuellus aniarus* | Otgun County, Zavkhan Province, Mongolia | 2019/8/6 | 2668 | 98.101 | 47.610 |  |
| *Mocuellus aniarus* | Otgun County, Zavkhan Province, Mongolia | 2019/8/6 | 2668 | 98.101 | 47.610 |  |
| *Mocuellus bulganica* | Otgun County, Zavkhan Province, Mongolia | 2019/8/6 | 2668 | 98.101 | 47.610 |  |
| *Mocuellus collina* | Otgun County, Zavkhan Province, Mongolia | 2019/8/7 | 2144 | 97.820 | 47.210 |  |
| *Mocuellus inepta* | Tsetserleg County, Arkhangai Province, Mongolia | 2019/8/2 | 1805 | 100.295 | 48.108 |  |
| *Mocuellus inepta* | Tsetserleg County, Arkhangai Province, Mongolia | 2019/8/2 | 1805 | 100.295 | 48.108 |  |
| *Mocuellus sibiricus* | Chair County, Arkhangai Province, Mongolia | 2019/8/3 | 2248 | 99.122 | 48.049 |  |
| *Mocuellus sibiricus* | Chair County, Arkhangai Province, Mongolia | 2019/8/3 | 2248 | 99.122 | 48.049 |  |
| *Mocuellus sibiricus* | Chair County, Arkhangai Province, Mongolia | 2019/8/3 | 2248 | 99.122 | 48.049 |  |
| *Ophiola corniculus* | Chair County, Arkhangai Province, Mongolia | 2019/8/4 | 2136 | 99.049 | 48.077 |  |
| *Ophiola jakowleffi* | Chair County, Arkhangai Province, Mongolia | 2019/8/3 | 2248 | 99.122 | 48.050 |  |
| *Ophiola transversa* | Chair County, Arkhangai Province, Mongolia | 2019/8/3 | 2248 | 99.122 | 48.050 |  |
| *Pinumius areatus* | Bayan Herkhan County, Zavkhan Province, Mongolia | 2019/8/6 | 2668 | 98.101 | 47.610 |  |
| *Pinumius areatus* | Bayan Herkhan County, Zavkhan Province, Mongolia | 2019/8/6 | 2668 | 98.101 | 47.610 |  |
| *Pinumius areatus* | Bayan Herkhan County, Zavkhan Province, Mongolia | 2019/8/6 | 2668 | 98.101 | 47.610 |  |
| *Pinumius areatus* | Bayan Herkhan County, Zavkhan Province, Mongolia | 2019/8/6 | 2668 | 98.101 | 47.610 |  |
| *Pinumius areatus* | Bayan Herkhan County, Zavkhan Province, Mongolia | 2019/8/6 | 2668 | 98.101 | 47.610 |  |
| *Pinumius areatus* | Bayanchandmani County,Tuv Province, Mongolia | 2019/8/1 | 1370 | 106.111 | 48.179 |  |
| *Pinumius areatus* | Tsetserleg County, Arkhangai Province, Mongolia | 2019/8/7 | 2149 | 97.620 | 47.240 |  |
| *Pinumius areatus* | Tsetserleg County, Arkhangai Province, Mongolia | 2019/8/7 | 2149 | 97.620 | 47.240 |  |
| *Pinumius areatus* | Tsetserleg County, Arkhangai Province, Mongolia | 2019/8/7 | 2149 | 97.620 | 47.240 |  |
| *Pinumius areatus* | Tsetserleg County, Arkhangai Province, Mongolia | 2019/8/7 | 2149 | 97.620 | 47.240 |  |
| *Pinumius areatus* | Tsetserleg County, Arkhangai Province, Mongolia | 2019/8/7 | 2149 | 97.620 | 47.240 |  |
| *Pinumius areatus* | Tsetserleg County, Arkhangai Province, Mongolia | 2019/8/7 | 2149 | 97.620 | 47.240 |  |
| *Pinumius areatus* | Tsetserleg County, Arkhangai Province, Mongolia | 2019/8/7 | 2149 | 97.620 | 47.240 |  |
| *Psammotettix alienulus* | Radarham County, Zavkhan Province, Mongolia | 2019/8/8 | 1748 | 96.823 | 47.736 |  |
| *Psammotettix confinis* | Radarham County, Zavkhan Province, Mongolia | 2019/8/8 | 1817 | 96.932 | 47.732 |  |
| *Psammotettix confins* | Radarham County, Zavkhan Province, Mongolia | 2019/8/8 | 1817 | 96.932 | 47.732 |  |
| *Psammotettix koeleriae* | Radarham County, Zavkhan Province, Mongolia | 2019/8/8 | 1817 | 96.932 | 47.732 |  |
| *Psammotettix mongolicus* | Tsetserleg County, Arkhangai Province, Mongolia | 2019/8/7 | 2380 | 97.112 | 47.215 |  |
| *Psammotettix mongolicus* | Chair County, Arkhangai Province, Mongolia | 2019/8/5 | 3005 | 98.246 | 47.687 |  |
| *Psammotettix mongolicus* | Chair County, Arkhangai Province, Mongolia | 2019/8/5 | 3005 | 98.246 | 47.687 |  |
| *Psammotettix nodosus* | Chair County, Arkhangai Province, Mongolia | 2019/8/5 | 2224 | 98.901 | 48.010 |  |
| *Stenometopiellus ersinicus* | Chair County, Arkhangai Province, Mongolia | 2019/8/3 | 2248 | 99.122 | 48.049 |  |
| *Stenometopiellus signativus* | Chair County, Arkhangai Province, Mongolia | 2019/8/3 | 2248 | 99.122 | 48.050 |  |
| *Stenometopiellus zavchanus* | Ulanhus County, Bayan-Ölgii Province, Mongolia | 2019/8/14 | 2707 | 88.098 | 49.185 |  |
| *Tiaratus caricis* | Bayan Herkhan County, Zavkhan Province | 2019/8/6 | 2224 | 98.901 | 48.010 |  |
| *Concavifer sagittatus* | - | - | - | - | - |  |
| *Emeljanovianus hilaris* | - | - | - | - | - |  |
| *Emeljanovianus hilaris* | - | - | - | - | - |  |
| *Emeljanovianus hilaris* | - | - | - | - | - |  |
| *Macrosteles guttatus* | - | - | - | - | - |  |
| *Drabescus ineffectus* | - | - | - | - | - |  |
| *Fieberiella septentrionalis* | - | - | - | - | - |  |
