# Supplementary material for: Phylogeographic and Ecological Insights Into the Evolutionary History of the Grass‐ and Sedge‐Specializing Deltocephalinae Leafhoppers
Source: Ecol Evol. 2026 Jan 5;16(1):e72857. doi: 10.1002/ece3.72857 (PMC12771680; doi:10.1002/ece3.72857)
Supplement: Supplementary file 2 — Table S2: Eight variables for the Middle Paleolithic‐Warm Period. [file ECE3-16-e72857-s002.docx]

| Variable | Environment factors |
| --- | --- |
| BIO1 | Annual Mean Temperature |
| BIO2 | Mean diurnal range |
| BIO3 | Isothermality |
| BIO4 | Temperature Seasonality |
| BIO14 | Precipitation of Driest Month |
| BIO15 | Precipitation seasonality |
| BIO16 | Precipitation of Wettest Quarter |
| BIO17 | Precipitation of direst quarter |
